# Disruption of hospital care during the first year of the COVID-19 pandemic impacted socioeconomic groups differently: population based study using routine registration data

**DOI:** 10.1186/s12913-024-10695-9

**Published:** 2024-03-06

**Authors:** Tessa Jansen, Sigur Gouwens, Lotta Meijerink, Iris Meulman, Lisanne H. J. A. Kouwenberg, G. Ardine de Wit, Johan J. Polder, Anton E. Kunst, Ellen Uiters

**Affiliations:** 1 Centre for Public Health, Healthcare, and Society, National Institute for Public Health and the Environment (RIVM), PO Box 1, 3720 BA Bilthoven, The Netherlands; 2 Tranzo, Tilburg School of Social and Behavioral Sciences, Tilburg University, 5000 LE Tilburg, The Netherlands; 3 Department of Public and Occupational Health, Amsterdam Public Health Research Institute, Amsterdam UMC, University of Amsterdam, 1105 BK Amsterdam, the Netherlands; 4 Department of Health Sciences, Faculty of Science & Amsterdam Public Health Research Institute, Vrije Universiteit Amsterdam, 1007 MB Amsterdam, The Netherlands; 5 Centre for Prevention, Lifestyle and Health, National Institute for Public Health and the Environment (RIVM), 3720 BA Bilthoven, The Netherlands

**Keywords:** COVID-19, Healthcare disparities, Socioeconomic factors, Health services

## Abstract

**Background:**

During the COVID-19 pandemic, provision of non-COVID healthcare was recurrently severely disrupted. The objective was to determine whether disruption of non-COVID hospital use, either due to cancelled, postponed, or forgone care, during the first pandemic year of COVID-19 impacted socioeconomic groups differently compared with pre-pandemic use.

**Methods:**

National population registry data, individually linked with data of non-COVID hospital use in the Netherlands (2017–2020). in non-institutionalised population of 25–79 years, in standardised household income deciles (1 = low, 10 = high) as proxy for socioeconomic status. Generic outcome measures included patients who received hospital care (dichotomous): outpatient contact, day treatment, inpatient clinic, and surgery. Specific procedures were included as examples of frequently performed elective and acute procedures, e.g.: elective knee/hip replacement and cataract surgery, and acute percutaneous coronary interventions (PCI). Relative risks (RR) for hospital use were reported as outcomes from generalised linear regression models (binomial) with log-link. An interaction term was included to assess whether income differences in hospital use during the pandemic deviated from pre-pandemic use.

**Results:**

Hospital use rates declined in 2020 across all income groups. With baseline (2019) higher hospital use rates among lower than higher income groups, relatively stronger declines were found for lower income groups. The lowest income groups experienced a 10% larger decline in surgery received than the highest income group (RR 0.90, 95% CI 0.87 – 0.93). Patterns were similar for inpatient clinic, elective knee/hip replacement and cataract surgery. We found small or no significant income differences for outpatient clinic, day treatment, and acute PCI.

**Conclusions:**

Disruption of non-COVID hospital use in 2020 was substantial across all income groups during the acute phases of the pandemic, but relatively stronger for lower income groups than could be expected compared with pre-pandemic hospital use. Although the pandemic’s impact on the health system was unprecedented, healthcare service shortages are here to stay. It is therefore pivotal to realise that lower income groups may be at risk for underuse in times of scarcity.

**Supplementary Information:**

The online version contains supplementary material available at 10.1186/s12913-024-10695-9.

## Introduction

During the COVID-19 pandemic, the healthcare system worldwide was recurrently severely disrupted by the influx of patients with COVID-19 [1, 2]. Provision of regular care was challenged by shortages of healthcare workers due to sick leave and quarantine [3]. Particularly during the first wave of infections in 2020 there was also a lack of resources, such as personal protective equipment and ventilators [3, 4]. On the supply side, non-COVID-19 elective care was downscaled by delaying or cancelling procedures to free capacity for COVID-19 patients [3]. On the demand side people forewent care, for instance because they were reluctant to seek care out of fear for SARS-CoV-2 infection, or not wanting to burden healthcare workers with ‘minor’ health problems [5–7]. During the first pandemic wave, a median reduction of about one third of healthcare services was found across 20 countries [8]. The volume of non-COVID related hospital admissions dropped substantially, with sharpest drops for elective admissions. However, also non-COVID-19 emergency admissions decreased [8–12]. In the Netherlands, during 2020 the total volume of hospital admissions decreased with 12% compared with 2019 [13]. Consequently, an estimated 320 thousand quality adjusted life years were lost due to postponed or forgone elective surgery in the Netherlands in 2020 and 2021 [9, 14, 15]. Disrupted hospital use in this study refers to hospital care that is either postponed or cancelled by the healthcare provider, or postponed or foregone by the patient. It was impossible to disentangle what proportions of care were delivered as planned, involved catching-up backlogs of postponed care, changed due to changed need, or dissolved altogether.

Concerns have been expressed that the pandemic both highlighted and possibly reinforced health inequalities. For instance through higher susceptibility to contract the SARS-CoV-2 virus, worse course of disease after infection, and stronger impact of containment measures due to unfavourable living and working conditions [16–18]. Whether disruption of care impacted socioeconomic groups differently is not yet inconclusively demonstrated. Signs of ‘missing patients’ who did not present during the pandemic and led to dissolved cancer and cardiovascular disease diagnoses suggest unmet need [10, 19]. This likely unmet need may have been larger among individuals with lower socioeconomic status (SES) due to higher prevalence of these diseases [20]. Disrupted provision of healthcare therefore may have affected existing pre-pandemic socioeconomic inequalities in healthcare use [21]. Additionally, the shifted care pathways and increased use of e-health may have disadvantaged individuals with limited health literacy, generally being low SES individuals [22, 23].

Previous studies show mixed findings regarding groups that have been impacted the most by disrupted care during the pandemic. On the one hand especially care for mild illnesses seems to be affected, however also emergency care faced substantial reductions [8]. In England, NHS registry data showed that the drop of hospital admissions was unevenly distributed across living areas [12, 24]. In more deprived areas, the decline in elective admissions was larger than in more affluent areas, whereas the decline in emergency admissions for low-severity health problems was smaller in more deprived areas [24]. In a sample of vulnerable patients in Switzerland, forgoing care was found to be related to younger age, women, lower education, and chronic illness [25]. In a US-sample, financial strain and worse self-reported health, but also higher education and higher income were found to be associated with both foregone and delayed care [26]. Similarly, both foregone or postponed care in a sample of older adults from 27 European countries was related to higher education, though without taking health status into account [27]. These studies either reported registration data on aggregated level or self-reported foregone care. The association of disrupted hospital care with individual level socioeconomic factors is not widely studied using routine registration data. Accordingly, in our study we aimed to quantify the extent to which disruption of hospital use during the first pandemic year impacted socioeconomic groups differently. We therefore compared deviations of hospital use during the pandemic with pre-pandemic use for distinct socioeconomic groups.

## Methods

### Study population

For this observational population based study we used population registry data of Statistics Netherlands [28] linked at individual level with routine registry data of hospital use from January 1, 2017 to December 31, 2020. Hospital data included insurance claims data from all hospitals and independent treatment centres (ITCs) in the Netherlands, from Vektis (https://vektis.nl). Data were made available in the secured remote access environment of Statistics Netherlands. Statistics Netherlands functioned as a trusted third party according to Dutch law (Statistics Netherlands Act 2003), enabling the linkage between datasets while ensuring the privacy of the involved individuals [28]. The study population consisted of individuals living in a non-institutionalised household, aged 25 to 79 years. We excluded institutionalised individuals and elderly individuals. For these groups, other care arrangements and other mechanisms than in the general population are likely to impact healthcare seeking as well. Examples are home care for community dwelling elderly or nursing home care [29]. Individuals who deceased during the year were included for the time they were alive.

We studied diverging hospital use patterns throughout the first pandemic year. Therefore, we identified four phases that were delineated by the level of virus transmission in the population and measures issued to mitigate the spread of the virus. We specified phases as follows:

- Phase I, initial worldwide cases: January (week 2) to early March (week 10);
- Phase II, first wave of infections: March (week 11) to June (week 24). In week 11, the WHO [30] declared the pandemic. This was followed by the first wave of cases and containment measures such as a partial lock-down. Containment measures were relaxed in week 24;
- Phase III, intermediate phase: June (week 25) to September (Week 37). This phase was characterised by low infection rates and relaxing of containment measures;
- Phase IV, second wave of infections: September (week 38) to end of the year (week 52). During the second wave, increasing infection rates were followed by gradually stricter containment measures.

To enable comparison of hospital use in the first pandemic year with previous years, we subdivided previous years according to the calendar periods delineated by the pandemic phases in 2020 throughout the analyses. Administrative weeks 1 and 53 of a calendar year, as used for registration purposes, may vary in number of days, hindering comparability of years (and phases). We therefore omitted week 1 and 53 from the analyses.

### Measures

#### Outcomes

We operationalised hospital use outcomes as care activities within a diagnosis-related group. These include a combination of diagnosis and treatment activities [31]. We distinguished between generic outcomes such as outpatient contacts, and specific (surgical) procedures. To capture the extent of the pandemic’s impact on different types of hospital care across socioeconomic groups, we included general non-health problems specific hospital care activities. We specified surgical procedures, outpatient contacts, day treatment, and inpatient clinic. Since it was impossible to disentangle COVID-related activities from other hospital use, we excluded pulmonary and internal medicine specialities for all types of care. By excluding these specialties, an estimated 0.5% of surgical procedures was lost (range 2017–2020: 0.40–0.45%), despite surgeries likely not involved care for COVID-19 patients [32].

To distinguish possible differences between socioeconomic groups in use of elective and acute care during the phases of high influx of COVID-patients, we included three specific frequently performed invasive procedures (Supplementary Table 1). Elective procedures included two types of procedures that could be postponed without immediate hazard: elective joint replacement surgery (knee/hip) for osteoarthritis, and cataract surgery. In addition, we included percutaneous coronary intervention (PCI) for acute myocardial infarction as example of an acute procedure that could not be postponed without substantial hazard. The latter included acute PCI only, semi-acute or elective PCI were excluded (Supplementary Table 1).

We operationalised hospital use as an individual who received hospital care (dichotomous, yes/no) by calendar period (phase and year) as identified before, within an outcome as specified before. Consequently, if an individual had two outpatient contacts in a given calendar period (phase), these were counted as one patient. Whereas an individual who had different procedures in the same calendar period (phase), the individual was counted once for each distinct procedure. For instance if someone had an inpatient clinic admission and a surgical procedure, the individual was counted once for both procedures.

#### Independent measures

We used income as indicator for socioeconomic status as it is the most widely available measure in Dutch population data. We operationalised income as net disposable household income, standardised for household size and composition at January 1st for each calendar year. We derived standardised household income from the System of Social Databases of Statistics Netherlands [28]. We categorised income in deciles based on the study population by year, ranging from 1 (low) to 10 (high). For knee/hip replacement surgery, cataract surgery and PCI, the income deciles were determined within the population of ≥ 50 to 79 years since these procedures are rarely undergone by younger age groups. Rescaling of the income groups was conducted to account for a possible age-effect in distribution of income groups that may have impacted the relative socioeconomic position within the age-limited population. We omitted individuals with missing income from the analyses. The percentage of the population with missing income was similar over all included years (approximately 0.55%).

We used additional SES indicators education and standardised household assets, as indicator for wealth, for sensitivity analyses and robustness of our findings. We derived these measures from the System of Social Databases of Statistics Netherlands [28]. We considered these measures less suitable for primary analyses. Specifically because education is missing for a substantial share of the population of 55 years and older, and assets particularly capture SES of older age groups since these are cumulated over the life course [33]. Highest attained educational level was categorised in three groups according to ISCED-classification [34] and a group with missing information regarding education: high (higher professional education, university), intermediate (intermediate or advanced general education, intermediate vocational education), and low (no education, primary school only, lower vocational education). The group with missing education was substantial and therefore was included in the models as distinct group. Assets based on tax-returns were included as standardised household possessions (e.g. savings, securities, and real estate) minus debts (e.g. principal residence loans, education loans), categorised in deciles.

#### Confounders/background characteristics

We included age in 5 year age-bands ranging from 25 to 79 years, derived from date of birth as recorded in the personal records database. For knee/hip replacement surgery, cataract surgery and PCI, the study population was restricted to ≥ 50 years.

Sex included female/male as registered in the personal records database.

### Statistical analysis

To describe the study sample, we used descriptive statistics. To show developments over time for each of the hospital outcomes, we calculated the number of patients who had at least one of the specified contacts or procedures by year and per 10,000 population. To obtain relative risks (RRs) for hospital use, we fitted generalised linear models (GLM) with a binomial family and log-link function for each pandemic phase in 2020 or similar calendar period in previous years. The models were specified as follows:

$$\begin{array}{c}{P}_{phase}=\,i\,(Y=y\,|\,year\,=\,j,\,agesex\,={agesex}_{k}\,,\,income\,=\,inc\_k)\\\,=\,exp\,({\beta }_{1j}+{\beta }_{2j}\,{inc}_{k}+\,{\beta}_{3j}\,{inc}_{k}\,{year}_{j}+\,{\beta}_{4j}\,{year}_{k}\,+\,{\beta}_{5j}\,{agesex}_{k}\,+\,{\beta}_{6j} {agesex}_{k}\,{year}_{j})\end{array}$$

where: i = 1, 2, 3, 4 represents the phase of the year; j = 2017, 2018, 2019, 2020 is the calendar year; k = 1, …, N (number of individuals) (individuals); y = 0, 1 (each outcome measure).

**Fig. 1:**
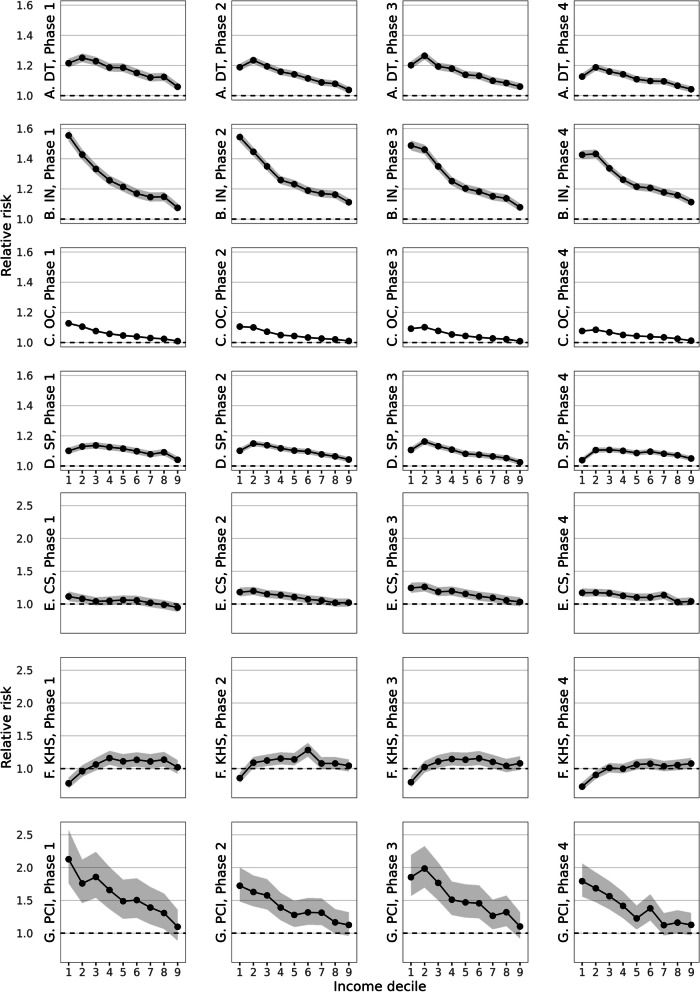
**a**-**g** Relative risk for hospital use in 2019 by income group and calendar phases for comparability with pandemic phases in 2020, with highest income group as reference for total population in the Netherlands of 25–79 years (**A**-**D**) or 50–79 years (**E**–**G**). Legend: panel **A** to **D** include generic outcomes (**A** DT: Day Treatment; **B** IN: Inpatient Clinic; **C** OC: Outpatient Clinic; **D** SP: Surgical Procedures). **E** to **G** include specific procedures (**E** CS: Cataract Surgery; **F** KHS: Knee or Hip replacement Surgery; **G** PCI: Percutaneous coronary intervention (PCI). Reference is highest income group 10, indicated by the dashed line at RR 1.0. RRs are age- and sex standardised. Note that the scaling of the Y-axis differs between **A**-**D** and **E**–**G**

**Fig. 2:**
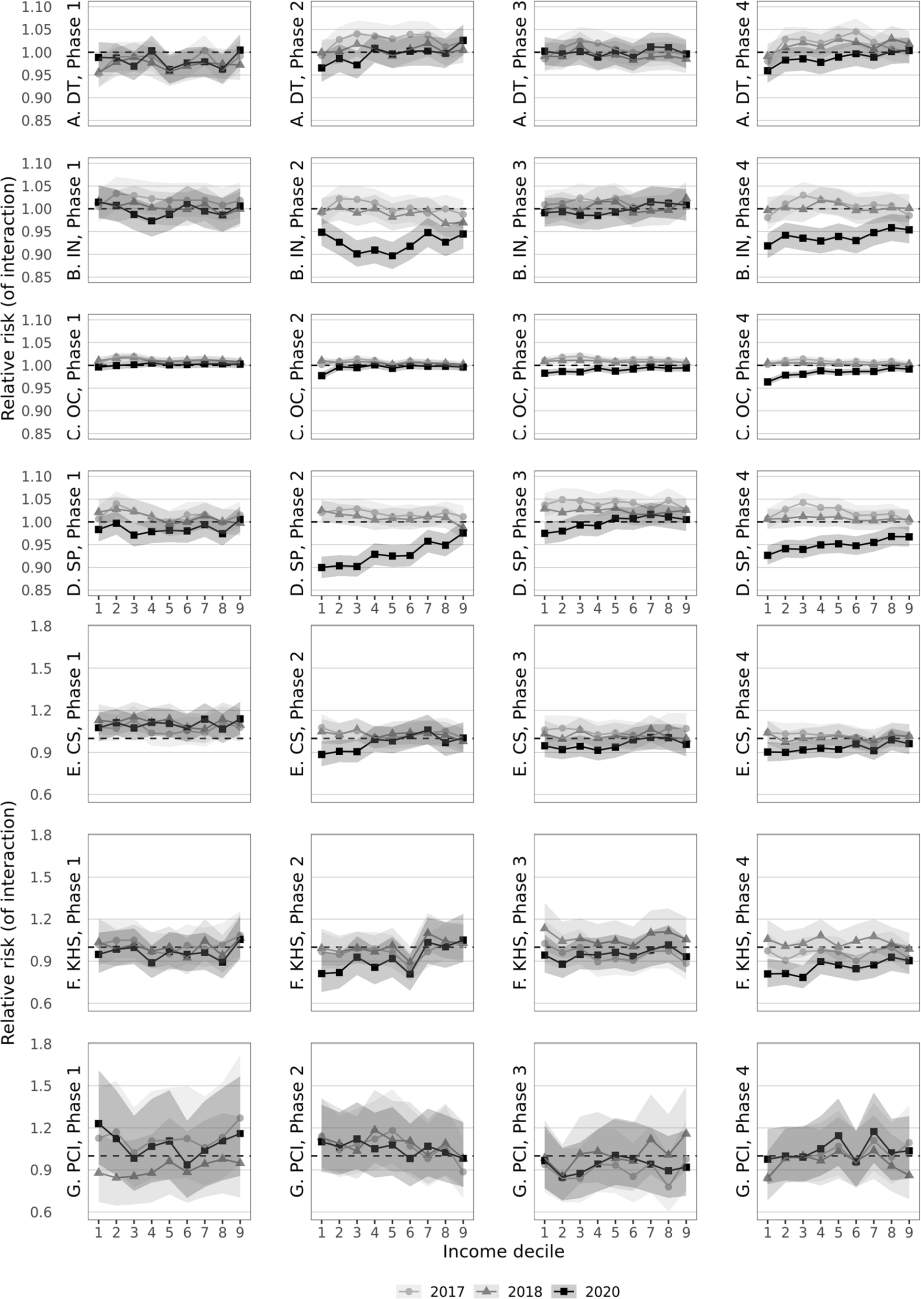
**a**-**g** Relative risk for hospital use for pandemic phases in 2020 (and calendar phases in 2017 and 2018) compared with differences in 2019 by income group, difference-in-difference with highest income group as reference for total population in the Netherlands of 25–79 years (**A**-**D**) or 50–79 years (**E**–**G**). Legend: **A** to **D** include generic outcomes (**A** DT: Day Treatment; **B** IN: Inpatient Clinic; **C** OC: Outpatient Clinic; **D** SP: Surgical Procedures). **E** to G include specific procedures (**E** CS: Cataract Surgery; **F** KHS: Knee or Hip replacement Surgery; **G** PCI: Percutaneous coronary intervention (PCI). Reference is highest income group 10, indicated by the dashed line at RR 1.0. RRs are age- and sex standardised. Note that the scaling of the Y-axis differs between **A**-**D** and **E**–**G**

The outcome *Ρ* denotes the probability to have used hospital care within each pandemic phase in 2020 or calendar period in previous years, specified for seven distinct outcomes. Taking surgery for example, the outcome is the probability that a person received at least one surgical procedure in the given pandemic phase. The term *β*_*2*_ × *income* decile captures income differences in healthcare use with the highest income group as reference. For instance, if the probability was 5% for the highest income group to have had surgery, the RRs of the other income groups indicate the deviation from that probability. To assess whether and to what extent year contributed to differences between income groups in hospital use compared with baseline year 2019, we included the interaction term *β*_*3*_ × *year* × *income decile* as main term of interest. We first plotted the exponentiated coefficients of baseline year 2019 as RRs by income group, with the highest income group as reference with RR fixed at 1.00 (Fig. 1, β2 when year = 2019). Subsequently, we plotted RRs for each pandemic phase and outcome, with the highest income decile and year 2019 set as reference with the RR fixed at 1.00 (Fig. 2, β3). These RRs should be interpreted as multiplicative to the baseline RRs in 2019. Accordingly, if healthcare use in 2019 were a pie that was divided in pieces for each income group, the differences in size of the pieces would be the RRs by income group compared with the highest income group. In 2020, the pie of healthcare use shrunk compared to 2019. The RRs from the interaction term include the effect of year for each income group. These RRs therefore refer to the change in size of portions of the pie for each income group compared with the share for the highest income group, within that smaller pie.

We added years 2017 to 2020 as dummy variables to the models, indicated by term *β*_*4*_ × *year*. We included years 2017 and 2018 in the models to compare with pre-pandemic years and therewith assess the size of natural fluctuations, additional to 2019 as reference year, and 2020 as pandemic year,. We included these years both in terms *β*_*3*_ and* β*_*4*_. If confidence intervals of 2020 did not overlap with previous years, we considered deviations as result from the pandemic instead of natural fluctuation. We adjusted all models for differences in hospital use patterns across age and sex groups for each year by an interaction term for sex and age-group × year.

Additionally, we conducted sensitivity analyses to assess the robustness of the associations between hospital use and SES. Therefore, we repeated all analyses with educational attainment and household assets respectively as alternative indicators for SES. Confidence intervals (CIs) were set at 95%. Analyses were conducted in R version 4.1.3 [35], using the GLM function from the stats-package (3.6.2).

## Results

### Characteristics of the study population

The study population consisted of approximately 11.5 million people in 2020 (almost 11.5 million in 2019), with similar distributions over age-groups in 2019 and 2020 (Supplementary Table 2). Table 1 displays hospital use per 10,000 population. Hospital use outcomes suggest different patterns over time, with declining inpatient clinic admissions and day treatment, and increasing outpatient contacts and surgery between 2017 and 2019. The pandemic year 2020 shows a decrease for all outcomes.

**Table 1:** Patients with hospital use, by 10,000 general population of 25–79 years, by year (2017–2020)

	Year			
	2017	2018	2019	2020
	n/10,000	n/10,000	n/10,000	n/10,000
**Generic outcomes**				
Outpatient contact	4,195	4,214	4,228	3,913
Day treatment	603	594	592	510
Inpatient clinic	593	569	558	485
Surgery	900	904	920	802
**Specific surgical procedures**				
Cataract surgery	88.2	92.5	94.7	83.0
Knee or hip replacement surgery	42.0	43.3	44.6	35.6
Percutaneous coronary intervention (PCI)	13.6	13.6	14.3	14.1

### Pre-pandemic differences in hospital use between socioeconomic groups

Figure 1 (panel a to g) depicts income differences (on the X-axis) for hospital use in 2019. The phases refer to distinct periods during the pandemic in 2020 since the models were fitted for each phase separately. For comparability with 2020 and to show that patterns naturally fluctuate during a year, RRs for 2019 were plotted by these phases as well. The plots show a generic pattern of higher relative risks (on the Y-axis) for hospital use for lower income groups, with the highest income group as reference (dashed line at RR 1.00). Largest income differences were found for acute PCI, with an RR of 1.72 for the lowest income group in calendar phase II (95% CI 1.57 – 1.87), compared with the highest income group. For surgery, income differences were less pronounced, with an RR of 1.10 (95% CI 1.09 – 1.12) for the lowest income compared with the highest income group in phase II. A dissimilar pattern was found for knee/hip replacement surgery. The lowest income group had a lower RR of 0.86 in phase II (95% CI 0.76 – 0.95) to receive a knee/hip replacement compared with the highest income group, whereas middle incomes had higher RRs than the highest income group. (Underlying RRs and 95% CIs from the plots are reported in Supplementary Table 3).

### Differences between socioeconomic groups in disrupted hospital use during the pandemic

Deviations in the income distribution of relative risk for hospital use compared with baseline income differences in 2019 are depicted in Fig. 2 (panel a to g). Deviations in 2020 (black line, squares) were considered to ensue from the pandemic if the confidence intervals of 2020 (shaded area around the plots) do not overlap with the reference line for 2019 (dashed line set at RR 1.00) and confidence intervals of 2017 and 2018. The overall decline of hospital use in 2020 compared with 2019 was set at RR 1.00 for the tenth income decile as highest income group. Deviations from RR 1.00 refer to larger or smaller declines in 2020 than could be expected based on income differences that existed in 2019. A relative risk of RR 0.90 may be interpreted as a 10% smaller probability to have received care than the reference group, as surplus on the decline that was experienced by the reference group. That is, adjusted for baseline income differences and within comparable sex and age-groups.

In phase I 2020, before the onset of the pandemic, for none of the hospital use outcomes significant deviations from previous years were found. From the outset of the pandemic, in phase II, declines in outpatient contacts were comparable across income groups, except for the lowest income group (RR 0.98, 95% CI 0.97 – 0.98). For surgical procedures and inpatient clinic admissions, declines were statistically significantly larger among lower income groups. Income groups one to three had an RR of 0.90 (95% CI 0.88 – 0.93), which may be interpreted as a 10% lower than usual (2019) probability to have received surgery. For inpatient clinic, income groups three to five had the lowest probabilities compared with pre-pandemic use.

During phase III, almost all care resembled use patterns of 2019. Somewhat lower probabilities for care use were found for outpatient contacts, but these were generic across income groups. An exception was found for surgical procedures, for which the lowest income group had a borderline significant 2.5% lower probability than in 2019 to have had surgery (RR 0.97, 95% CI 0.95 – 1.00).

In phase IV, during the second SARS-CoV-2 wave, patterns were comparable with phase II, though deviations from 2019 were somewhat smaller. The lowest income group had a 3.5% smaller probability to have had an outpatient contact (RR 0.96, 95% CI 0.96 – 0.97) and a 8% smaller probability for an inpatient clinic admission (RR 0.92, 95% CI 0.89 – 0.95). For surgery the lowest income group had the lowest probability compared with the highest income group (RR 0.93, 95% CI 0.90 – 0.95), although differences with other income groups were smaller than in phase II.

Zooming in at the specific procedures (Fig. 2, panel e–g), a similar pattern is shown for phase II and IV for planned cataract and knee/hip replacement surgery compared with surgery in total. Although differences in RRs between income groups were more distinct than for overall surgery, confidence intervals were large due to smaller numbers of patients. The lowest two income groups had an approximately 20% lower probability than in 2019, compared with the highest income group to have had knee or hip replacement surgery (income group one, phase II: RR 0.81, 95% CI 0.63 – 0.99, Table 2; phase IV: RR 0.81, 95% CI 0.68 – 0.93). For cataract surgery in phase II, the three lowest income groups had a lower probability than the highest income group compared with 2019 (lowest income group: RR 0.89, 95% CI 0.78 – 0.98). In phase IV, income groups in the lowest half all had approximately 10% lower probabilities than the highest income group to have had cataract surgery than could be expected from use patterns in 2019 (lowest income group: RR 0.90, 95% CI 0.83 – 0.98). During all phases in 2020, no significant income differences were found for acute PCI, indicating that the share of hospital care use for PCI across income groups remained stable. (Underlying RRs and 95% CIs from the plots for 2020 are reported in Supplementary Table 4). Table 2 shows an exemplar snapshot of Supplementary Tables 3 and 4 including RR’s and 95% CI’s for knee or hip replacement surgery, comparing 2019 and 2020.

**Table 2:** Snapshot of Relative risks for knee or hip replacement surgery in pandemic phase 2 in 2019 (corresponding with Fig. 1, panel F, phase 2) and 2020 (as deviation from 2019, corresponding with Fig. 2, panel F, phase 2), by income group for total population in the Netherlands within age-bands, standardised for age and sex

year	2019	2020, deviating from 2019
	RR (95% CI)	RR (95% CI)
**Phase 2**		
Income decile 1	0.86 (0.76 -0.95)	0.81 (0.63 -0.99)
Income decile 2	1.09 (1.01 -1.17)	0.82 (0.67 -0.97)
Income decile 3	1.12 (1.04 -1.20)	0.93 (0.78 -1.08)
Income decile 4	1.15 (1.07 -1.23)	0.86 (0.70 -1.01)
Income decile 5	1.14 (1.06 -1.22)	0.92 (0.76 -1.07)
Income decile 6	1.28 (1.20 -1.36)	0.81 (0.65 -0.96)
Income decile 7	1.08 (0.99 -1.16)	1.03 (0.88 -1.19)
Income decile 8	1.08 (0.99 -1.17)	1.00 (0.84 -1.16)
Income decile 9	1.04 (0.96 -1.13)	1.05 (0.89 -1.21)
Income decile 10	ref	ref

Comparison with the years 2017 and 2018, shows higher use rates for outpatient clinic and day treatment in 2017, compared with baseline year 2019. There were no significant differences in income patterns compared with income differences in 2019. Sensitivity analyses with education (Supplementary Figure 1, panel a – g) and household assets (Supplementary Figure 2, panel a – g) showed similar patterns.

## Discussion

### Principal findings

The aim of this study was to determine whether the disruption of hospital use in 2020, after the pandemic outbreak of COVID-19, was generic or impacted socioeconomic groups differently. Whereas virtually all hospital care was downscaled at the onset of the pandemic, the effect on lower income groups was larger than for higher income groups. Although the total volume of hospital care delivered declined for all income groups (a smaller pie), the portioning of the shares across income groups changed compared with pre-pandemic use. Shares of lower income groups declined more than those of higher income groups (smaller piece of the smaller pie). Particularly during phases of increasing infection rates (phase II and IV) the lowest income groups used smaller proportions of surgery than could be expected based on pre-pandemic use compared with the highest income groups. Comparison between specific procedures elective knee/hip surgery and cataract surgery versus acute PCI suggest that income differences in surgical procedures may have emerged from stronger disruption of elective surgery among lower income groups, whereas acute procedures were performed ‘as usual’. We found small or no statistically significant changes between income groups in the proportions of care use for outpatient clinic contacts, day treatment, and acute PCI.

### Strengths and limitations

This study encompassed nearly all specialised hospital care in the Netherlands, therefore its representativeness for the Dutch population and large power are particular strengths of this study. Furthermore, availability of multiple years of hospital care data enabled robust comparison of pre-pandemic socioeconomic differences with changes during the pandemic. In interpreting the results however, a number of limitations have to be taken into account. Firstly, health status is an important explanation for socioeconomic differences in healthcare use [21]. However, we did not have an encompassing measure to appropriately account for the patient’s health status. That may have induced bias if the relation between health status and healthcare use was different during the pandemic compared with previous years. Secondly, the structure of administrative hospital data does not allow to distinguish (patterns of) substitution between different types of care. Therefore, it is possible that surgical procedures to some extent have been substituted by outpatient contacts, or by treatment in primary care. If this was prevalent in certain SES-groups due to differences in complexity of care need, this may have biased the results. Particularly during phase II however, substitution potential in primary care was limited, as considerable decreases in primary care use were observed as well [6, 36]. Thirdly, excess mortality due to COVID-19 was not at random, but higher among lower income groups [37–39]. Their higher probability to receive hospital care in pre-pandemic times therefore may have been bypassed by prematurely dying from COVID-19 during the second wave [40], whereas during the first wave COVID-19 mortality substituted other-cause mortality among lower income groups [39]. Moreover, the higher probability of contracting SARS-CoV-2 for lower SES groups yielded additional competing risk for not using hospital care for elective surgery when infected, or already being in hospital due to COVID-19 infection. We were unable to distinguish COVID-19 patients in the data that we had at our disposal. Consequently, both higher COVID-19 related mortality and morbidity among lower income groups may have underestimated their probability of hospital care use in 2020. Lastly, our findings should be interpreted as variation on the national level. On the sub-national level however, regional differences in spread of the virus and capacity agreements within and between hospitals likely impacted differences between income groups.

### Comparison with existing literature

As discussed before, previous studies show mixed results regarding the populations that experienced disrupted healthcare [24–27]. Several mechanisms may underly our findings that low SES individuals experienced stronger declines in particularly surgical procedures compared with pre-pandemic use. We were unable to disentangle supply side and demand side factors, such as underlying motives and decisions of medical staff to cancel, postpone or substitute care, and from patients to forego care.

From the demand side, health literacy may have played a role in the decision of patients to forego care. Furthermore, health literate individuals may be better able to evaluate whether their health problem needed attention, acquire priority due to their ability to navigate through the healthcare system, for instance using waiting list referral services from their health insurance, interact with healthcare providers, and persistence in healthcare seeking [22, 23]. As health literacy is strongly correlated with SES this may partly explain why low SES individuals experienced stronger declines in certain types of care.

From the supply side, clinical decisions to treat someone in hospital may well have turned out differently for socioeconomic groups due to differences in health status [21]. Health status may have affected clinical decisions in different directions. If individuals who were most at risk of worsening health were prioritised [8, 10], that should have been reflected in higher care use among low SES individuals due to their generally poorer health [21]. Contrastingly, those with better health (and generally higher SES) may have been prioritised because they put lower strain on limited healthcare capacity due to smaller likelihood to develop complications (with the risk of ICU admission) [41].

Our findings concerning elective knee/hip replacement and cataract surgery suggest that allocation of mainly elective procedures benefitted patients who needed uncomplicated routine care. Patients in good health who needed knee or hip replacement for instance, may have been eligible for treatment in an independent treatment centre, were treatment was continued during the surge of COVID-19 patients in hospital [10]. Consequently, the repercussions of unmet healthcare need may have disproportionately impacted individuals with vulnerable health [42]. Moreover, their situation may have worsened while waiting for treatment [43]. Eventually, a cascading effect of worsening health due to delayed care may have hindered eligibility for surgery at all [43].

### Implications for research and practice

The pandemic is likely to cast a long shadow ahead in the repercussions of disrupted hospital care. Similar to the extending elective waiting lists in the UK [44, 45], the backlog of disrupted care in the Netherlands has accumulated even more in the second pandemic year with successive surges of COVID-19 [46]. Despite periods of low COVID-care demand, until mid-2022 it was seemingly impossible to accelerate care provision, thus waiting lists persisted [46]. Nevertheless, the pandemic merely reinforced pre-existing health workforce shortages by high turnover and sick leave during the pandemic [3, 10]. Accordingly, scarcity of workforce will be the default mode and emphasises the need for awareness that vulnerable groups may be overlooked. A cue may be found in the observation that part of the care demand seems to have been vanished [8, 11]. It yields the debate of what care is essential [8] and when substitution by, for instance, primary care or by e-health is appropriate [10, 47]. In that respect it is pivotal to tailor health services to patients’ abilities and needs and closely monitor whether care provision does not yield unwarranted inequalities and unmet need in specific groups [10, 19].

## Conclusion

The unprecedented disruption of hospital care during the first year of the COVID-19 pandemic impacted socioeconomic groups differently. Our findings show that lower income groups relatively received smaller proportions of hospital care than could be expected from pre-pandemic use patterns and this varied for specific types of care. Undoubtedly, the COVID-19 pandemic highlighted emerging challenges of the healthcare system’s capacity and the need for pandemic preparedness. Accordingly, awareness regarding equitable care allocation is essential.

### Supplementary Information

**Supplementary material 1.**

## Data Availability

Results are based on calculations by the Dutch National Institute for Public Health and the Environment using non-public microdata from Statistics Netherlands. For further information regarding the use of this data for scientific research: microdata@cbs.nl. The data that support the findings of this study are available from Statistics Netherlands but restrictions apply to the availability of these data, which were used under license for the current study, and so are not publicly available. Data are however available from the corresponding author upon reasonable request and with permission of Statistics Netherlands. The unpublished statistical code is available upon reasonable request from the corresponding author as well.

## Abbreviations

CI: Confidence interval
COVID-19: Coronavirus Disease 2019
GLM: General linear model
ITC: Independent treatment centre
PCI: Percutaneous coronary interventions
RR: Relative risk
SARS-CoV-2: Severe acute respiratory syndrome coronavirus 2
SES: Socioeconomic status
