# Supplementary material for: Disruption of hospital care during the first year of the COVID-19 pandemic impacted socioeconomic groups differently: population based study using routine registration data
Source: BMC Health Serv Res. 2024 Mar 6;24:294. doi: 10.1186/s12913-024-10695-9 (PMC10918870; doi:10.1186/s12913-024-10695-9)
Supplement: Supplementary file 1 — Supplementary material 1. [file 12913_2024_10695_MOESM1_ESM.docx]

## Supplementary material

Supplementary table 1: Selection of specific procedures from diagnosis-related groups.

Supplementary table 1. Selection of hospital care activities from diagnosis-related groups

| Procedure | Specialty | Diagnoses | Care product | Care activity |
| --- | --- | --- | --- | --- |
| Cataract surgery | 301 (Ophthalmology) | 554 (Cataract | 70401009  70401002  70401008 | 31242  31241  31251  31268 |
| Percutaneous coronary intervention (for acute myocardial infarction) | 320 (Cardiology) | Independent from diagnosis | Independent care product | 33238 |
| Hip replacement (for arthrosis/arthritis) | 305 (Orthopaedics) | 1701 (Arthrosis pelvis/hip/ femur) | 131999052 | 38567 |
| Knee replacement (for arthrosis/arthritis) | 305 (Orthopaedics) | 1801 (Arthrosis knee) | 131999104 | 38663 |

Supplementary table 2: characteristics of the study population by year.

Supplementary table 2. Characteristics of the study population at January 1, 2019 and 2020

| Year | 2019 | 2020 |
| --- | --- | --- |
| Population (n) | 11,473,175 | 11,569,197 |
|  | % | % |
| Sex (female) | 50.3 | 50.3 |
| Age-group |  |  |
| 25-29 years | 9.7 | 9.7 |
| 30-34 years | 9.3 | 9.4 |
| 35-39 years | 8.9 | 8.9 |
| 40-44 years | 8.9 | 8.7 |
| 45-49 years | 10.7 | 10.2 |
| 50-54 years | 10.8 | 11.0 |
| 55-59 years | 10.7 | 10.5 |
| 60-64 years | 9.5 | 9.6 |
| 65-69 years | 8.6 | 8.5 |
| 70-74 years | 7.9 | 8.1 |
| 75-79 years | 5.2 | 5.4 |
| Education |  |  |
| Missing | 40.1 | 38.8 |
| Low | 12.8 | 12.8 |
| Intermediate | 23.2 | 23.7 |
| High | 24.0 | 24.7 |

Supplementary table 3: Relative risks (RRs) for hospital use in 2019 by income group, with highest income group as reference for total population in the Netherlands within age-bands.

Supplementary table 3. Relative risks for hospital use in 2019 by income group and calendar periods corresponding with pandemic phases in 2020 for total population in the Netherlands within age-bands, adjusted for age and sex

| Care activity | Outpatient contact  (25-79 years) | Day treatment  (25-79 years) | Inpatient clinic  (25-79 years) | Surgery  (25-79 years) | Cataract surgery  (50-79 years) | Knee or hip replacement surgery  (50-79 years) | Percutaneous coronary intervention (PCI)  (50-79 years) |
| --- | --- | --- | --- | --- | --- | --- | --- |
|  | RR (95% CI) | RR (95% CI) | RR (95% CI) | RR (95% CI) | RR (95% CI) | RR (95% CI) | RR (95% CI) |
|  |  |  |  |  |  |  |  |
| **Phase 1** |  |  |  |  |  |  |  |
| Income decile 1 | 1.13 (1.12 -1.13 ) | 1.22 (1.19 -1.24) | 1.55 (1.53 -1.58) | 1.10 (1.08 -1.12) | 1.11 (1.05 -1.18) | 0.78 (0.67 -0.88) | 2.13 (1.94 -2.32) |
| Income decile 2 | 1.11 (1.10 -1.11 ) | 1.25 (1.23 -1.27) | 1.43 (1.40 -1.45) | 1.13 (1.11 -1.15) | 1.08 (1.02 -1.14) | 0.96 (0.87 -1.05) | 1.76 (1.57 -1.95) |
| Income decile 3 | 1.08 (1.07 -1.08 ) | 1.23 (1.21 -1.25) | 1.33 (1.31 -1.36) | 1.14 (1.12 -1.15) | 1.04 (0.98 -1.10) | 1.06 (0.97 -1.15) | 1.86 (1.67 -2.04) |
| Income decile 4 | 1.06 (1.05 -1.06 ) | 1.19 (1.16 -1.21) | 1.26 (1.23 -1.28) | 1.12 (1.11 -1.14) | 1.05 (0.99 -1.11) | 1.16 (1.07 -1.25) | 1.66 (1.46 -1.85) |
| Income decile 5 | 1.05 (1.04 -1.05 ) | 1.19 (1.16 -1.21) | 1.21 (1.19 -1.24) | 1.12 (1.10 -1.13) | 1.06 (1.00 -1.13) | 1.11 (1.02 -1.21) | 1.49 (1.29 -1.69) |
| Income decile 6 | 1.04 (1.03 -1.05 ) | 1.15 (1.13 -1.17) | 1.17 (1.14 -1.20) | 1.10 (1.08 -1.12) | 1.06 (0.99 -1.12) | 1.13 (1.04 -1.23) | 1.51 (1.31 -1.70) |
| Income decile 7 | 1.03 (1.02 -1.04 ) | 1.12 (1.10 -1.14) | 1.15 (1.12 -1.17) | 1.08 (1.06 -1.10) | 1.02 (0.95 -1.08) | 1.11 (1.01 -1.21) | 1.39 (1.19 -1.59) |
| Income decile 8 | 1.02 (1.02 -1.03 ) | 1.12 (1.10 -1.15) | 1.15 (1.12 -1.17) | 1.09 (1.07 -1.11) | 0.99 (0.92 -1.06) | 1.14 (1.04 -1.23) | 1.31 (1.10 -1.51) |
| Income decile 9 | 1.01 (1.00 -1.02 ) | 1.06 (1.03 -1.08) | 1.07 (1.05 -1.10) | 1.04 (1.02 -1.06) | 0.95 (0.88 -1.02) | 1.02 (0.92 -1.12) | 1.10 (0.88 -1.31) |
| Income decile 10 | ref | ref | ref | ref | ref | ref | ref |
| **Phase 2** |  |  |  |  |  |  |  |
| Income decile 1 | 1.11 (1.10 -1.11) | 1.19 (1.17 -1.21) | 1.54 (1.52 -1.56) | 1.10 (1.08 -1.12) | 1.18 (1.12 -1.24) | 0.86 (0.76 -0.95) | 1.72 (1.57 -1.87) |
| Income decile 2 | 1.10 (1.10 -1.10) | 1.23 (1.22 -1.25) | 1.45 (1.43 -1.47) | 1.15 (1.13 -1.16) | 1.20 (1.15 -1.25) | 1.09 (1.01 -1.17) | 1.63 (1.48 -1.77) |
| Income decile 3 | 1.07 (1.07 -1.08) | 1.19 (1.18 -1.21) | 1.35 (1.33 -1.37) | 1.14 (1.12 -1.15) | 1.15 (1.10 -1.20) | 1.12 (1.04 -1.20) | 1.58 (1.43 -1.72) |
| Income decile 4 | 1.05 (1.04 -1.05) | 1.16 (1.14 -1.18) | 1.26 (1.24 -1.28) | 1.12 (1.10 -1.13) | 1.14 (1.08 -1.19) | 1.15 (1.07 -1.23) | 1.39 (1.24 -1.54) |
| Income decile 5 | 1.04 (1.04 -1.05) | 1.14 (1.12 -1.16) | 1.23 (1.21 -1.25) | 1.10 (1.09 -1.12) | 1.11 (1.05 -1.16) | 1.14 (1.06 -1.22) | 1.28 (1.12 -1.43) |
| Income decile 6 | 1.03 (1.03 -1.04) | 1.11 (1.09 -1.13) | 1.19 (1.17 -1.21) | 1.10 (1.08 -1.11) | 1.07 (1.01 -1.13) | 1.28 (1.20 -1.36) | 1.32 (1.16 -1.47) |
| Income decile 7 | 1.03 (1.02 -1.03) | 1.09 (1.07 -1.11) | 1.17 (1.15 -1.19) | 1.08 (1.06 -1.09) | 1.05 (1.00 -1.11) | 1.08 (0.99 -1.16) | 1.31 (1.16 -1.47) |
| Income decile 8 | 1.02 (1.02 -1.03) | 1.08 (1.06 -1.10) | 1.16 (1.14 -1.18) | 1.06 (1.05 -1.08) | 1.02 (0.96 -1.08) | 1.08 (0.99 -1.17) | 1.17 (1.01 -1.32) |
| Income decile 9 | 1.01 (1.01 -1.02) | 1.04 (1.02 -1.06) | 1.11 (1.09 -1.13) | 1.04 (1.03 -1.06) | 1.02 (0.96 -1.08) | 1.04 (0.96 -1.13) | 1.13 (0.97 -1.28) |
| Income decile 10 | ref | ref | ref | ref | ref | ref | ref |
| **Phase 3** |  |  |  |  |  |  |  |
| Income decile 1 | 1.09 (1.09 -1.10) | 1.20 (1.18 -1.22) | 1.49 (1.47 -1.51) | 1.11 (1.09 -1.12) | 1.25 (1.18 -1.31) | 0.79 (0.69 -0.90) | 1.85 (1.69 -2.02) |
| Income decile 2 | 1.10 (1.10 -1.11) | 1.26 (1.24 -1.28) | 1.46 (1.44 -1.48) | 1.16 (1.15 -1.18) | 1.26 (1.21 -1.32) | 1.02 (0.94 -1.11) | 1.99 (1.83 -2.14) |
| Income decile 3 | 1.08 (1.07 -1.08) | 1.19 (1.17 -1.21) | 1.35 (1.33 -1.37) | 1.13 (1.11 -1.15) | 1.19 (1.13 -1.24) | 1.11 (1.02 -1.19) | 1.77 (1.60 -1.93) |
| Income decile 4 | 1.05 (1.05 -1.06) | 1.18 (1.16 -1.20) | 1.25 (1.23 -1.27) | 1.11 (1.09 -1.13) | 1.20 (1.14 -1.25) | 1.15 (1.06 -1.24) | 1.51 (1.34 -1.68) |
| Income decile 5 | 1.04 (1.04 -1.05) | 1.14 (1.12 -1.16) | 1.20 (1.18 -1.23) | 1.08 (1.06 -1.10) | 1.15 (1.09 -1.22) | 1.14 (1.04 -1.23) | 1.47 (1.30 -1.64) |
| Income decile 6 | 1.03 (1.03 -1.04) | 1.13 (1.11 -1.15) | 1.18 (1.16 -1.21) | 1.07 (1.06 -1.09) | 1.12 (1.06 -1.18) | 1.16 (1.06 -1.25) | 1.46 (1.28 -1.63) |
| Income decile 7 | 1.03 (1.02 -1.03) | 1.10 (1.08 -1.12) | 1.15 (1.13 -1.17) | 1.06 (1.05 -1.08) | 1.09 (1.03 -1.16) | 1.10 (1.01 -1.20) | 1.27 (1.09 -1.44) |
| Income decile 8 | 1.02 (1.02 -1.03) | 1.08 (1.06 -1.11) | 1.14 (1.11 -1.16) | 1.05 (1.03 -1.07) | 1.06 (0.99 -1.12) | 1.04 (0.95 -1.14) | 1.32 (1.14 -1.50) |
| Income decile 9 | 1.01 (1.00 -1.01) | 1.06 (1.04 -1.08) | 1.08 (1.05 -1.10) | 1.02 (1.01 -1.04) | 1.03 (0.97 -1.10) | 1.08 (0.98 -1.18) | 1.10 (0.92 -1.28) |
| Income decile 10 | ref | ref | ref | ref | ref | ref | ref |
| **Phase 4** |  |  |  |  |  |  |  |
| Income decile 1 | 1.08 (1.07 -1.08) | 1.13 (1.11 -1.15) | 1.43 (1.41 -1.45) | 1.04 (1.02 -1.05) | 1.17 (1.12 -1.23) | 0.73 (0.64 -0.81) | 1.79 (1.65 -1.93) |
| Income decile 2 | 1.08 (1.08 -1.09) | 1.19 (1.17 -1.21) | 1.43 (1.41 -1.45) | 1.11 (1.09 -1.12) | 1.17 (1.13 -1.22) | 0.90 (0.83 -0.97) | 1.68 (1.55 -1.82) |
| Income decile 3 | 1.07 (1.06 -1.07) | 1.16 (1.14 -1.18) | 1.34 (1.32 -1.36) | 1.11 (1.09 -1.12) | 1.16 (1.12 -1.21) | 1.01 (0.94 -1.08) | 1.56 (1.43 -1.70) |
| Income decile 4 | 1.05 (1.05 -1.06) | 1.14 (1.12 -1.16) | 1.26 (1.24 -1.28) | 1.10 (1.09 -1.12) | 1.13 (1.07 -1.18) | 1.00 (0.92 -1.07) | 1.42 (1.27 -1.56) |
| Income decile 5 | 1.04 (1.04 -1.05) | 1.11 (1.09 -1.13) | 1.21 (1.19 -1.24) | 1.09 (1.07 -1.10) | 1.10 (1.05 -1.15) | 1.06 (0.99 -1.14) | 1.23 (1.08 -1.37) |
| Income decile 6 | 1.04 (1.03 -1.04) | 1.10 (1.08 -1.12) | 1.21 (1.19 -1.23) | 1.10 (1.08 -1.11) | 1.10 (1.05 -1.15) | 1.07 (1.00 -1.15) | 1.38 (1.23 -1.52) |
| Income decile 7 | 1.03 (1.03 -1.04) | 1.09 (1.08 -1.11) | 1.18 (1.16 -1.20) | 1.08 (1.07 -1.10) | 1.14 (1.08 -1.19) | 1.04 (0.96 -1.11) | 1.12 (0.97 -1.27) |
| Income decile 8 | 1.03 (1.02 -1.03) | 1.07 (1.05 -1.09) | 1.16 (1.14 -1.18) | 1.07 (1.06 -1.09) | 1.03 (0.97 -1.09) | 1.05 (0.98 -1.13) | 1.16 (1.01 -1.31) |
| Income decile 9 | 1.01 (1.01 -1.02) | 1.04 (1.02 -1.06) | 1.11 (1.09 -1.13) | 1.05 (1.03 -1.06) | 1.04 (0.98 -1.10) | 1.08 (1.00 -1.15) | 1.13 (0.98 -1.28) |
| Income decile 10 | ref | ref | ref | ref | ref | ref | ref |

Supplementary table 4: Relative risks (RRs) for hospital use in 2020 as deviation from 2019, by income group, with highest income group as reference for total population in the Netherlands within age-bands

Supplementary table 4. Relative risks for hospital use in 2020 as deviation from 2019 by income group and pandemic phase for total population in the Netherlands within age-bands, adjusted for age and sex.

| Care activity | Outpatient contact  (25-79 years) | Day treatment (25-79 years) | Inpatient clinic (25-79 years) | Surgery (25-79 years) | Cataract surgery  (50-79 years) | Knee or hip replacement surgery  (50-79 years) | Percutaneous coronary intervention (PCI)  (50-79 years) |
| --- | --- | --- | --- | --- | --- | --- | --- |
|  | RR (95% CI) | RR (95% CI) | RR (95% CI) | RR (95% CI) | RR (95% CI) | RR (95% CI) | RR (95% CI) |
|  |  |  |  |  |  |  |  |
| **Phase 1** |  |  |  |  |  |  |  |
| Income decile 1 | 1.00 (0.99 -1.00) | 0.99 (0.96 -1.02) | 1.01 (0.98 -1.05) | 0.98 (0.96 -1.01) | 1.08 (0.98 -1.17) | 0.95 (0.80 -1.10) | 1.23 (0.96 -1.50) |
| Income decile 2 | 1.00 (0.99 -1.01) | 0.99 (0.96 -1.02) | 1.01 (0.97 -1.04) | 1.00 (0.97 -1.02) | 1.11 (1.03 -1.20) | 0.98 (0.86 -1.11) | 1.12 (0.86 -1.39) |
| Income decile 3 | 1.00 (0.99 -1.01) | 0.97 (0.94 -1.00) | 0.99 (0.95 -1.02) | 0.97 (0.94 -1.00) | 1.07 (0.99 -1.16) | 1.00 (0.87 -1.13) | 0.98 (0.71 -1.25) |
| Income decile 4 | 1.01 (1.00 -1.01) | 1.00 (0.97 -1.04) | 0.97 (0.94 -1.01) | 0.98 (0.95 -1.00) | 1.11 (1.03 -1.20) | 0.89 (0.76 -1.02) | 1.07 (0.79 -1.34) |
| Income decile 5 | 1.00 (0.99 -1.01) | 0.96 (0.93 -0.99) | 0.99 (0.95 -1.02) | 0.98 (0.95 -1.01) | 1.11 (1.02 -1.20) | 0.97 (0.84 -1.10) | 1.11 (0.82 -1.39) |
| Income decile 6 | 1.00 (0.99 -1.01) | 0.98 (0.94 -1.01) | 1.01 (0.97 -1.05) | 0.98 (0.95 -1.01) | 1.07 (0.98 -1.16) | 0.95 (0.81 -1.08) | 0.94 (0.65 -1.23) |
| Income decile 7 | 1.00 (0.99 -1.01) | 0.98 (0.95 -1.01) | 0.99 (0.96 -1.03) | 0.99 (0.97 -1.02) | 1.14 (1.04 -1.23) | 0.96 (0.83 -1.10) | 1.04 (0.75 -1.33) |
| Income decile 8 | 1.00 (0.99 -1.01) | 0.96 (0.93 -1.00) | 0.99 (0.95 -1.02) | 0.97 (0.95 -1.00) | 1.07 (0.97 -1.17) | 0.89 (0.75 -1.03) | 1.11 (0.81 -1.40) |
| Income decile 9 | 1.00 (0.99 -1.01) | 1.00 (0.97 -1.04) | 1.01 (0.97 -1.04) | 1.01 (0.98 -1.03) | 1.14 (1.04 -1.24) | 1.06 (0.91 -1.20) | 1.16 (0.86 -1.46) |
| Income decile 10 | ref | ref | ref | ref | ref | ref | ref |
| **Phase 2** |  |  |  |  |  |  |  |
| Income decile 1 | 0.98 (0.97 -0.98) | 0.97 (0.93 -1.00) | 0.95 (0.92 -0.98) | 0.90 (0.87 -0.93) | 0.89 (0.79 -0.99) | 0.81 (0.63 -0.99) | 1.10 (0.89 -1.32) |
| Income decile 2 | 1.00 (0.99 -1.00) | 0.99 (0.96 -1.02) | 0.93 (0.90 -0.96) | 0.90 (0.88 -0.93) | 0.91 (0.82 -1.00) | 0.82 (0.67 -0.97) | 1.06 (0.86 -1.27) |
| Income decile 3 | 0.99 (0.99 -1.00) | 0.97 (0.94 -1.00) | 0.90 (0.87 -0.93) | 0.90 (0.88 -0.93) | 0.91 (0.82 -1.00) | 0.93 (0.78 -1.08) | 1.12 (0.91 -1.33) |
| Income decile 4 | 1.00 (0.99 -1.01) | 1.01 (0.98 -1.04) | 0.91 (0.88 -0.94) | 0.93 (0.90 -0.95) | 0.99 (0.90 -1.08) | 0.86 (0.70 -1.01) | 1.05 (0.83 -1.27) |
| Income decile 5 | 0.99 (0.99 -1.00) | 1.00 (0.96 -1.03) | 0.90 (0.86 -0.93) | 0.92 (0.90 -0.95) | 0.98 (0.89 -1.08) | 0.92 (0.76 -1.07) | 1.08 (0.85 -1.30) |
| Income decile 6 | 1.00 (0.99 -1.01) | 1.00 (0.97 -1.03) | 0.92 (0.88 -0.95) | 0.93 (0.90 -0.95) | 1.02 (0.92 -1.12) | 0.81 (0.65 -0.96) | 0.98 (0.76 -1.21) |
| Income decile 7 | 1.00 (0.99 -1.01) | 1.00 (0.97 -1.04) | 0.95 (0.91 -0.98) | 0.96 (0.93 -0.98) | 1.06 (0.96 -1.16) | 1.03 (0.88 -1.19) | 1.07 (0.85 -1.29) |
| Income decile 8 | 1.00 (0.99 -1.01) | 1.00 (0.96 -1.03) | 0.93 (0.89 -0.96) | 0.95 (0.92 -0.98) | 0.97 (0.87 -1.07) | 1.00 (0.84 -1.16) | 1.03 (0.79 -1.26) |
| Income decile 9 | 1.00 (0.99 -1.00) | 1.03 (0.99 -1.06) | 0.94 (0.91 -0.98) | 0.98 (0.95 -1.00) | 1.00 (0.90 -1.11) | 1.05 (0.89 -1.21) | 0.98 (0.75 -1.22) |
| Income decile 10 | ref | ref | ref | ref | ref | ref | ref |
| **Phase 3** |  |  |  |  |  |  |  |
| Income decile 1 | 0.98 (0.98 -0.99) | 1.00 (0.97 -1.03) | 0.99 (0.96 -1.02) | 0.97 (0.95 -1.00) | 0.95 (0.86 -1.04) | 0.94 (0.80 -1.09) | 0.97 (0.74 -1.20) |
| Income decile 2 | 0.99 (0.98 -0.99) | 1.00 (0.97 -1.03) | 0.99 (0.96 -1.02) | 0.98 (0.96 -1.00) | 0.92 (0.84 -1.00) | 0.88 (0.76 -1.00) | 0.85 (0.63 -1.07) |
| Income decile 3 | 0.99 (0.98 -0.99) | 1.00 (0.97 -1.03) | 0.99 (0.95 -1.02) | 0.99 (0.97 -1.02) | 0.94 (0.86 -1.02) | 0.95 (0.83 -1.07) | 0.88 (0.65 -1.10) |
| Income decile 4 | 0.99 (0.99 -1.00) | 0.99 (0.96 -1.02) | 0.99 (0.95 -1.02) | 0.99 (0.97 -1.02) | 0.91 (0.83 -1.00) | 0.94 (0.82 -1.07) | 0.94 (0.71 -1.17) |
| Income decile 5 | 0.99 (0.98 -0.99) | 1.00 (0.97 -1.03) | 0.99 (0.96 -1.03) | 1.01 (0.98 -1.03) | 0.94 (0.85 -1.02) | 0.96 (0.84 -1.09) | 1.00 (0.77 -1.24) |
| Income decile 6 | 0.99 (0.98 -1.00) | 0.99 (0.96 -1.02) | 1.00 (0.97 -1.03) | 1.01 (0.98 -1.03) | 0.99 (0.90 -1.08) | 0.93 (0.81 -1.06) | 0.98 (0.75 -1.22) |
| Income decile 7 | 1.00 (0.99 -1.00) | 1.01 (0.98 -1.04) | 1.02 (0.98 -1.05) | 1.02 (0.99 -1.04) | 1.01 (0.92 -1.10) | 0.98 (0.85 -1.11) | 0.94 (0.69 -1.19) |
| Income decile 8 | 0.99 (0.99 -1.00) | 1.01 (0.98 -1.04) | 1.01 (0.98 -1.05) | 1.01 (0.99 -1.04) | 1.01 (0.91 -1.10) | 1.02 (0.88 -1.15) | 0.89 (0.65 -1.14) |
| Income decile 9 | 0.99 (0.99 -1.00) | 1.00 (0.96 -1.03) | 1.01 (0.97 -1.04) | 1.01 (0.98 -1.03) | 0.96 (0.86 -1.05) | 0.93 (0.80 -1.06) | 0.92 (0.67 -1.17) |
| Income decile 10 | ref | ref | ref | ref | ref | ref | ref |
| **Phase 4** |  |  |  |  |  |  |  |
| Income decile 1 | 0.96 (0.96 -0.97) | 0.96 (0.93 -0.99) | 0.92 (0.89 -0.95) | 0.93 (0.90 -0.95) | 0.90 (0.83 -0.98) | 0.81 (0.68 -0.93) | 0.98 (0.78 -1.18) |
| Income decile 2 | 0.98 (0.97 -0.99) | 0.98 (0.96 -1.01) | 0.94 (0.91 -0.97) | 0.94 (0.92 -0.96) | 0.90 (0.83 -0.97) | 0.81 (0.71 -0.92) | 1.00 (0.81 -1.19) |
| Income decile 3 | 0.98 (0.97 -0.99) | 0.99 (0.96 -1.01) | 0.94 (0.91 -0.96) | 0.94 (0.92 -0.96) | 0.92 (0.85 -0.99) | 0.78 (0.68 -0.89) | 0.99 (0.80 -1.19) |
| Income decile 4 | 0.99 (0.98 -0.99) | 0.98 (0.95 -1.00) | 0.93 (0.90 -0.96) | 0.95 (0.93 -0.97) | 0.93 (0.86 -1.00) | 0.90 (0.79 -1.00) | 1.05 (0.85 -1.25) |
| Income decile 5 | 0.98 (0.98 -0.99) | 0.99 (0.96 -1.02) | 0.94 (0.91 -0.97) | 0.95 (0.93 -0.97) | 0.92 (0.85 -1.00) | 0.87 (0.77 -0.98) | 1.14 (0.94 -1.35) |
| Income decile 6 | 0.99 (0.98 -0.99) | 1.00 (0.97 -1.02) | 0.93 (0.90 -0.96) | 0.95 (0.93 -0.97) | 0.96 (0.89 -1.03) | 0.85 (0.74 -0.95) | 0.96 (0.75 -1.16) |
| Income decile 7 | 0.99 (0.98 -0.99) | 0.99 (0.96 -1.02) | 0.95 (0.92 -0.98) | 0.96 (0.93 -0.98) | 0.91 (0.84 -0.99) | 0.87 (0.76 -0.98) | 1.17 (0.96 -1.39) |
| Income decile 8 | 0.99 (0.99 -1.00) | 1.00 (0.97 -1.03) | 0.96 (0.93 -0.99) | 0.97 (0.95 -0.99) | 0.99 (0.91 -1.07) | 0.93 (0.82 -1.04) | 1.02 (0.81 -1.23) |
| Income decile 9 | 0.99 (0.98 -1.00) | 1.00 (0.98 -1.03) | 0.95 (0.92 -0.99) | 0.97 (0.95 -0.99) | 0.96 (0.88 -1.04) | 0.90 (0.79 -1.01) | 1.04 (0.82 -1.25) |
| Income decile 10 | ref | ref | ref | ref | ref | ref | ref |

Supplementary figure 1.a-g.: plots RR hospital healthcare educational differences in 2020, adjusted for sex-age group × year. Note: the scaling of the Y-axis differs between panel A-D and E-G. Bars instead of lines to account for the category ‘missing’.

Supplementary figure 1.a-g. Relative risk for hospital healthcare use for pandemic phases in 2020 (and similar phases in 2017 and 2018) by educational group, difference-in-difference with highest educational group as reference for total population in the Netherlands of 25-79 years (panel A-D) or 50-79 years (panel E-G).

| a | 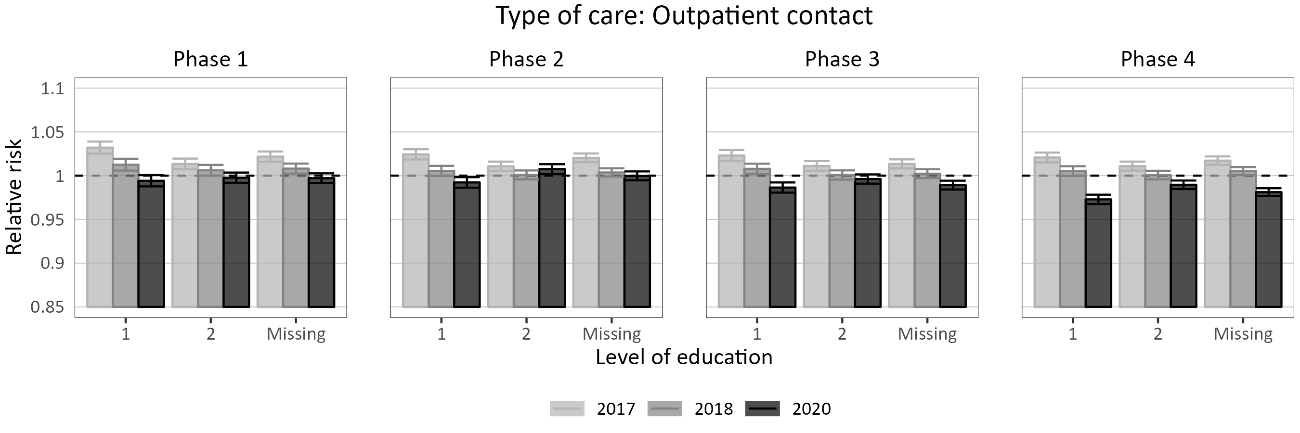 |
| --- | --- |
| b | 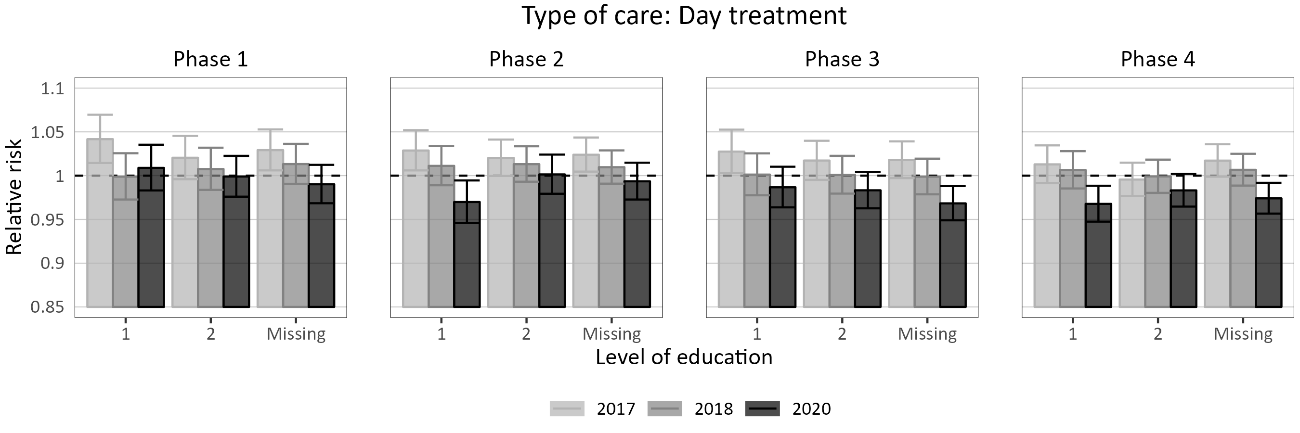 |
| c | 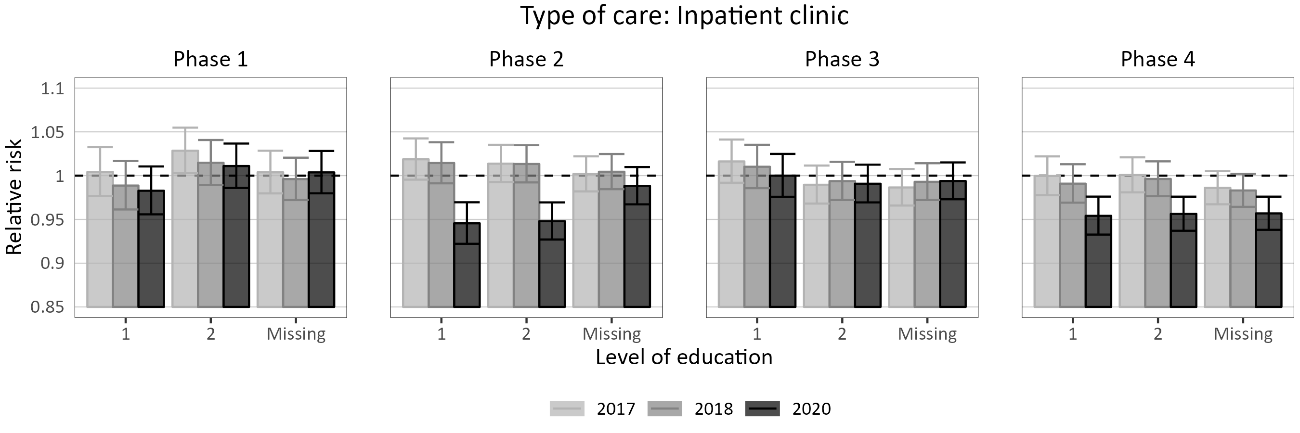 |
| d | 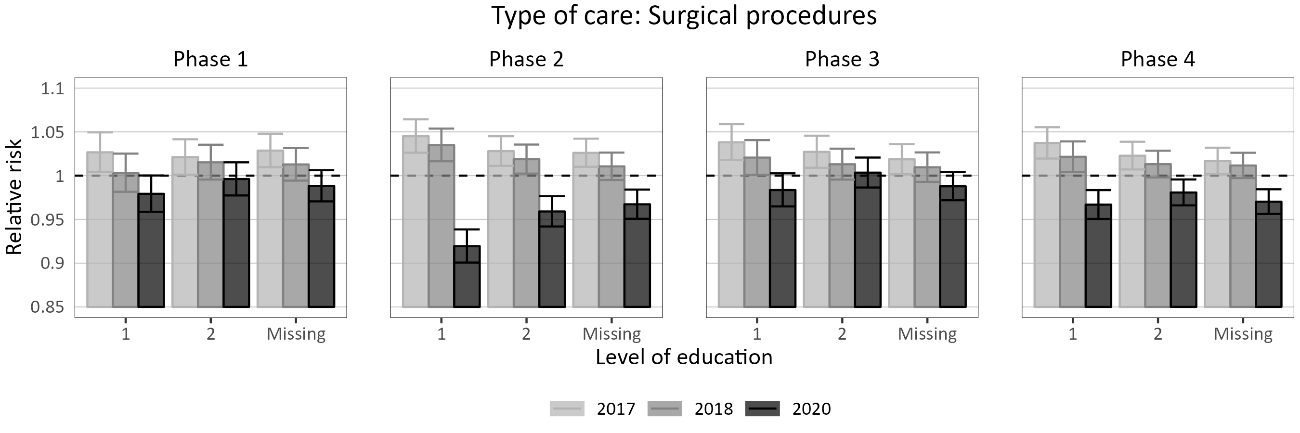 |
| e | 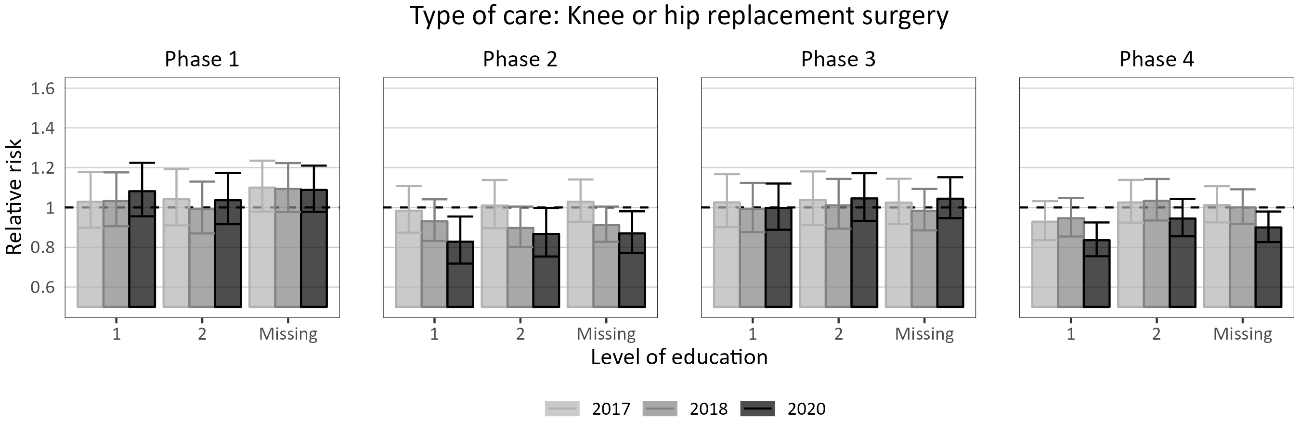 |
| f | 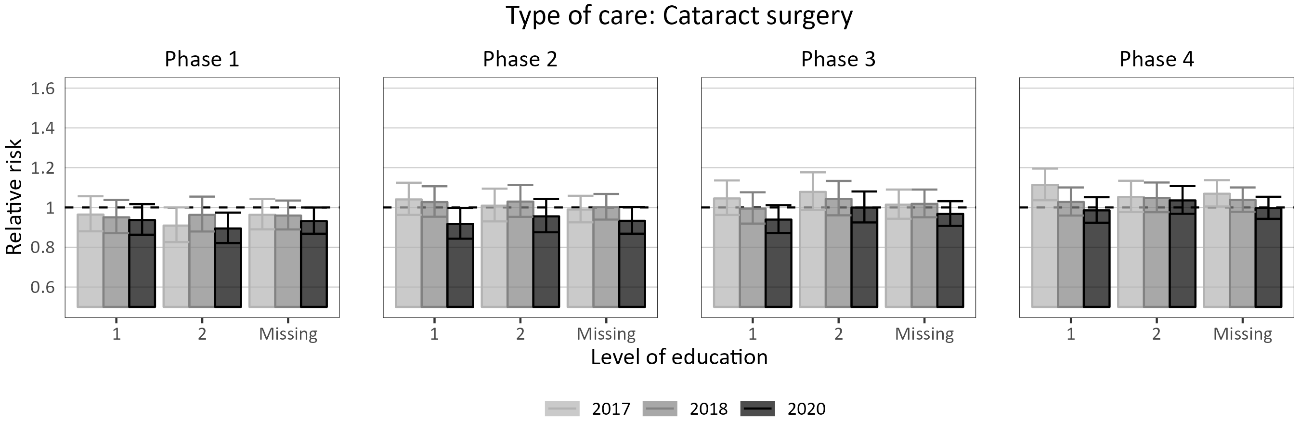 |
| g | 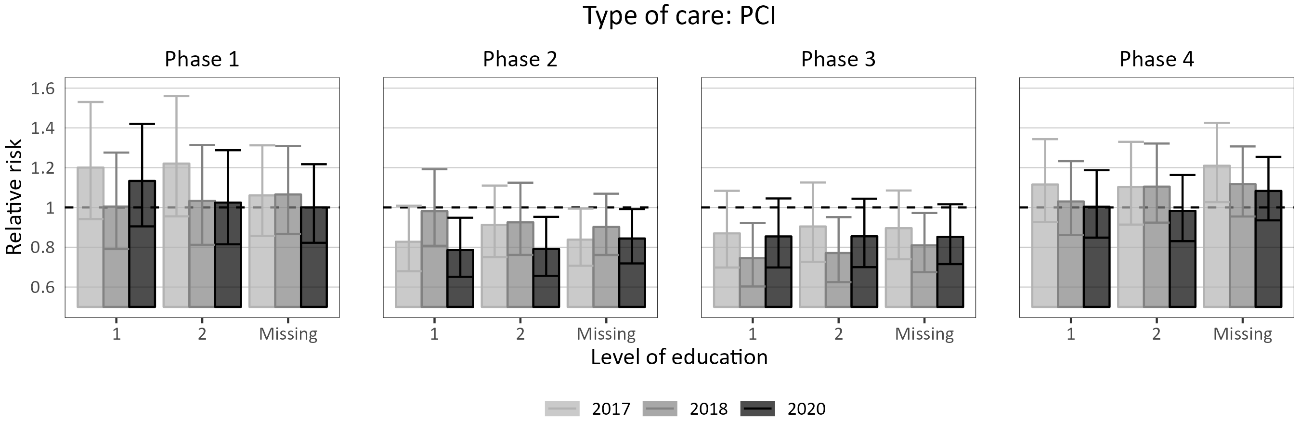 |

Legend: panel A to D include generic outcomes (A. DT: Day Treatment; B. IN: Inpatient Clinic; C. OC: Outpatient Clinic; D. SP: Surgical Procedures). Panel E to G include specific procedures (E. CS: Cataract Surgery; F. KHS: Knee or Hip replacement Surgery; G. PCI: Percutaneous coronary intervention (PCI). Reference is highest income group 10, indicated by the dashed line at RR 1.0. RRs are age- and sex adjusted.

Supplementary figure 2.a-g.: plots RR hospital healthcare differences assets groups in 2020, adjusted for age and sex. Note: the scaling of the Y-axis differs between panel A-D and E-G.

Supplementary figure 2.a-g. Relative risk for hospital healthcare use for pandemic phases in 2020 (and similar phases in 2017 and 2018) by assets group, difference-in-difference with highest assets group as reference for total population in the Netherlands of 25-79 years (panel A-D) or 50-79 years (panel E-G).

| a | 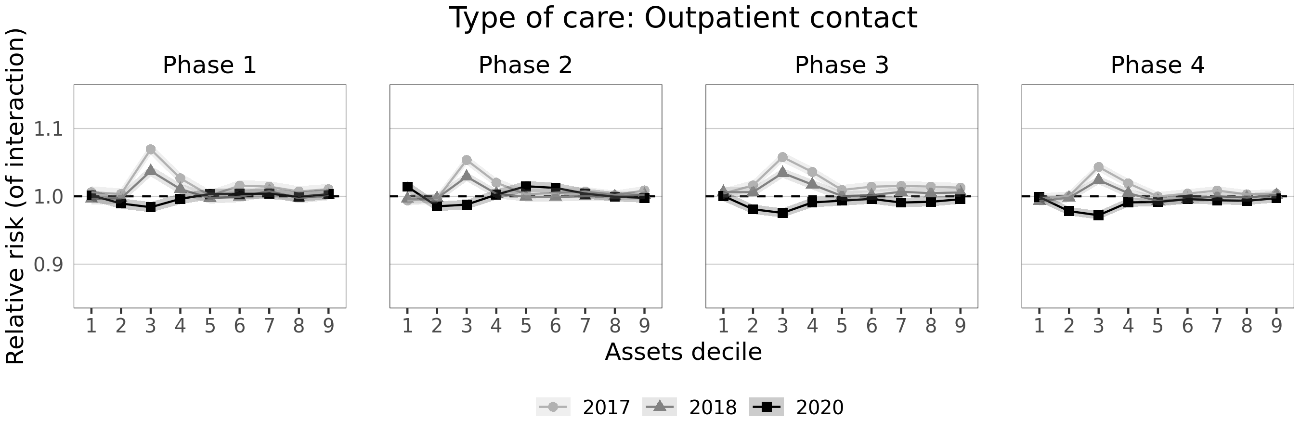 |
| --- | --- |
| b | 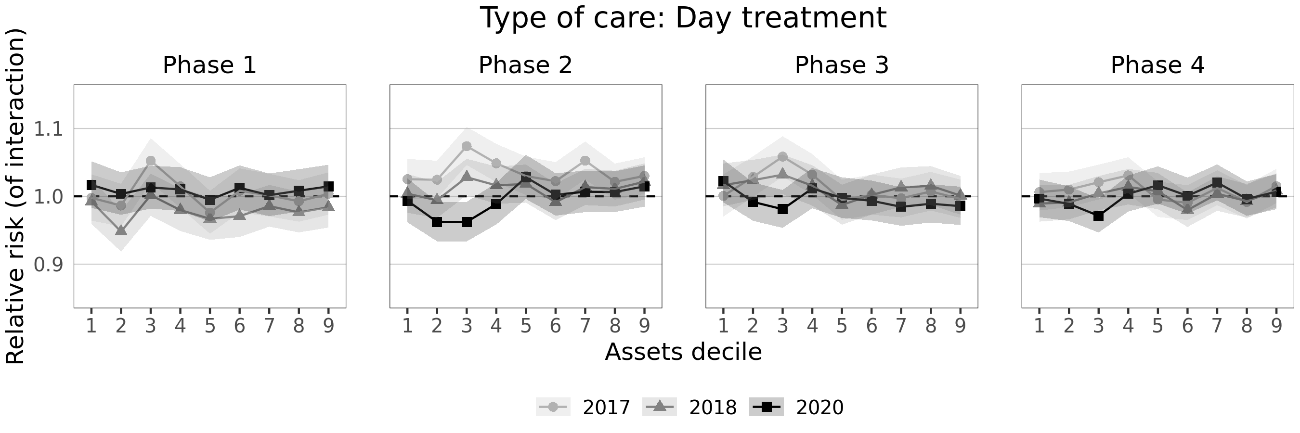 |
| c | 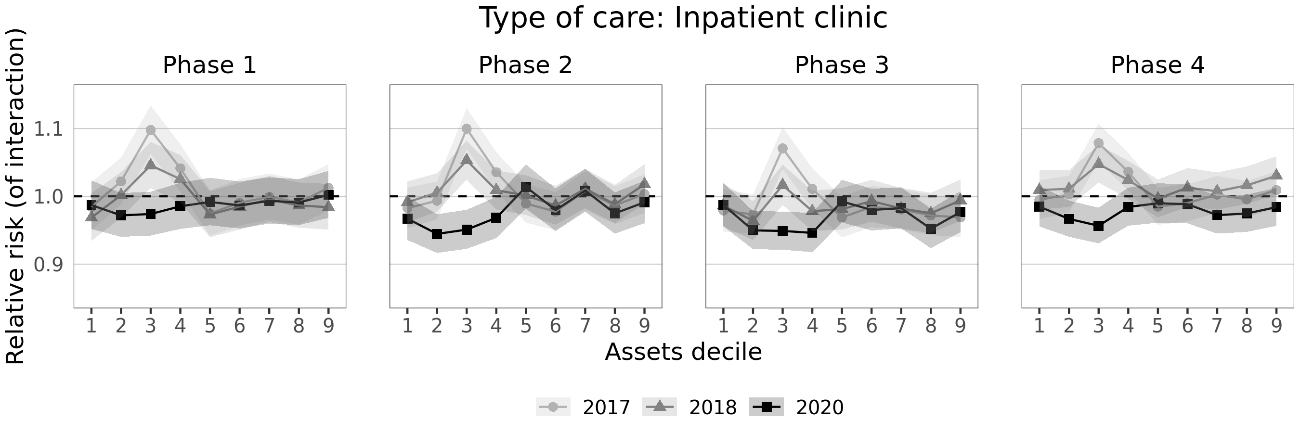 |
| d | 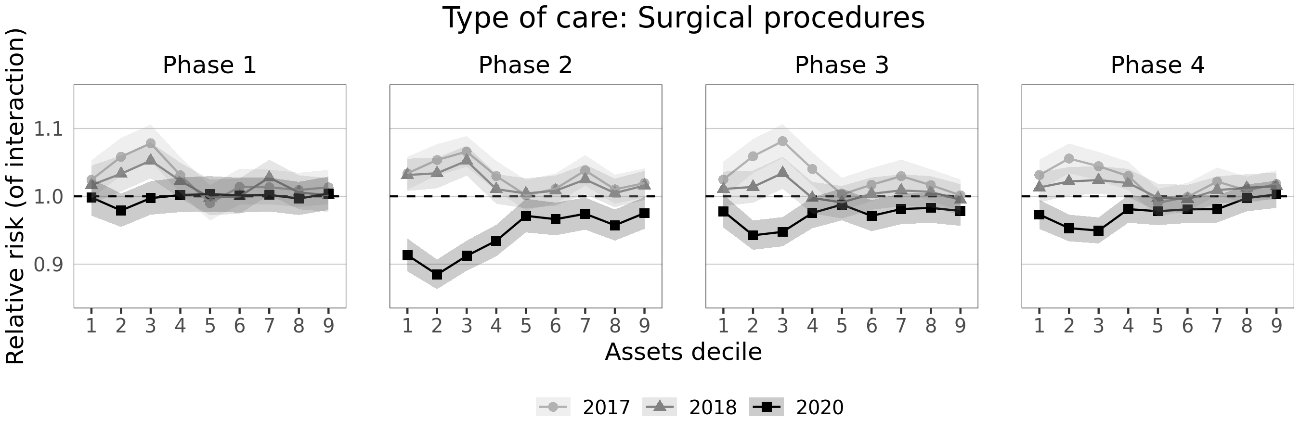 |
| e | 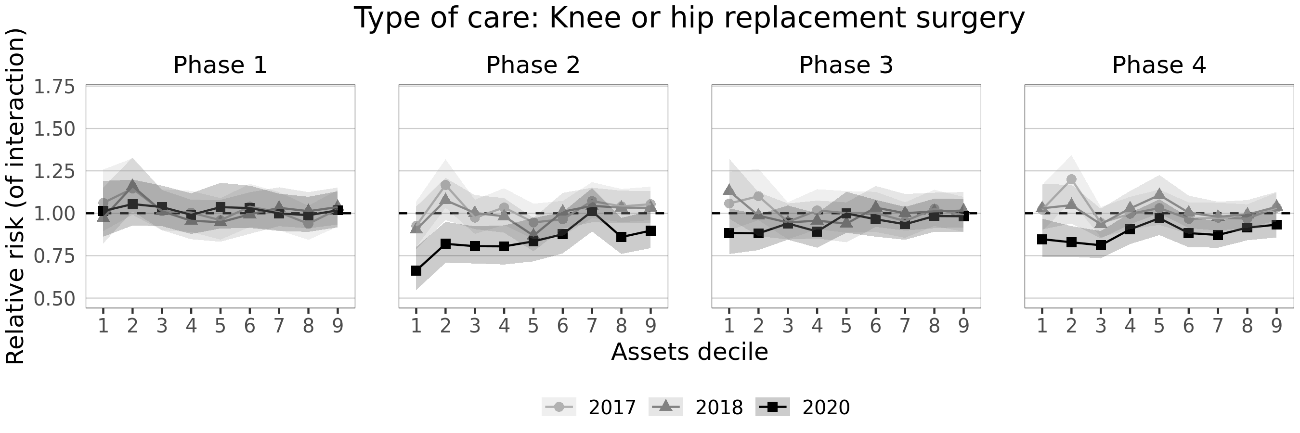 |
| f | 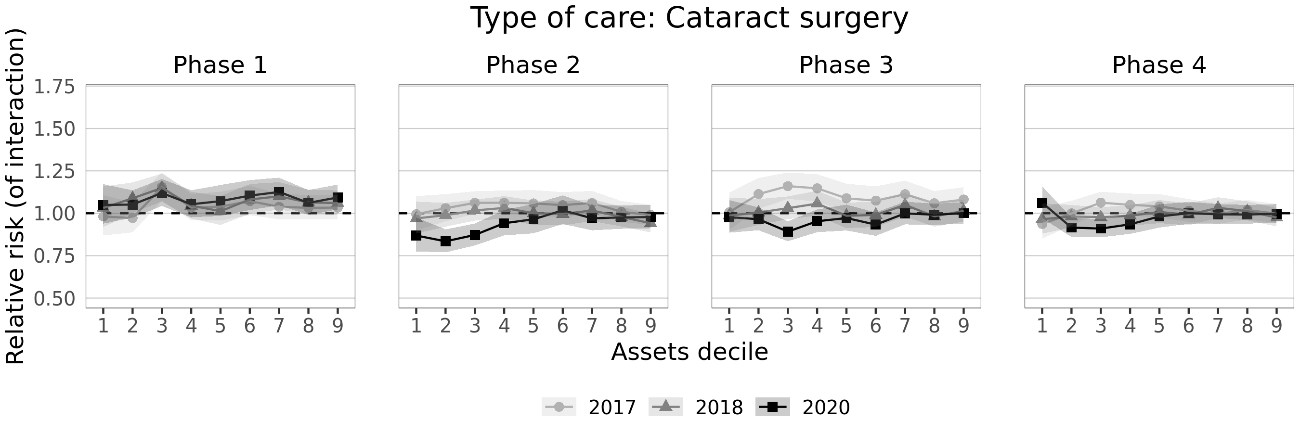 |
| g | 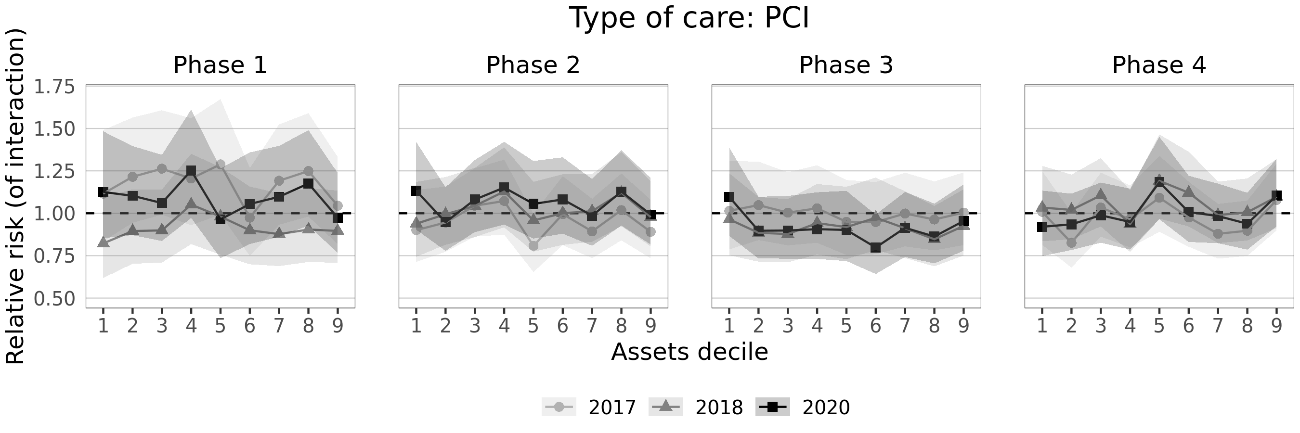 |

Legend: panel A to D include generic outcomes (A. DT: Day Treatment; B. IN: Inpatient Clinic; C. OC: Outpatient Clinic; D. SP: Surgical Procedures). Panel E to G include specific procedures (E. CS: Cataract Surgery; F. KHS: Knee or Hip replacement Surgery; G. PCI: Percutaneous coronary intervention (PCI). Reference is highest income group 10, indicated by the dashed line at RR 1.0. RRs are age- and sex adjusted.
